# A Novel Preclinical Murine Model to Monitor Inflammatory Breast Cancer Tumor Growth and Lymphovascular Invasion

**DOI:** 10.3390/cancers15082261

**Published:** 2023-04-12

**Authors:** Ashlyn G. Rickard, Dorababu S. Sannareddy, Alexandra Bennion, Pranalee Patel, Scott J. Sauer, Douglas C. Rouse, Samantha Bouchal, Harrison Liu, Mark W. Dewhirst, Gregory M. Palmer, Gayathri R. Devi

**Affiliations:** 1 Program of Medical Physics, Duke University, Durham, NC 27705, USA; 2 Department of Radiation Oncology, Duke University School of Medicine, Durham, NC 27710, USA; 3 Division of Surgical Sciences, Department of Surgery, Duke University School of Medicine, Durham, NC 27710, USA; 4 Trinity College of Arts and Sciences, Duke University, Durham, NC 27705, USA; 5 Division of Laboratory Animal Resources, Duke University School of Medicine, Durham, NC 27710, USA; 6 Duke Inflammatory Breast Cancer Consortium, Duke Cancer Institute, Durham, NC 27710, USA; 7 Program in Cancer Risk, Detection, and Interception, Duke Cancer Institute, Durham, NC 27710, USA

**Keywords:** window chamber, inflammatory breast cancer (IBC), tumor microenvironment, lymphatic vessels, migration, SUM149

## Abstract

**Simple Summary:**

Lymphovascular invasion (LVI), the presence of tumor cells in lymphovascular spaces, is associated with an increased risk of metastasis and is considered an independent prognostic indicator. LVI is a clinicopathological hallmark of inflammatory breast cancer (IBC), an understudied but highly lethal variant presenting as diffuse tumor cell clusters (tumor emboli) invading throughout the breast and dermal lymphatics, causing breast erythema and edema and often without a distinct tumor mass. There is a lack of preclinical IBC models that monitor spatial and temporal changes during growth and migration of individual tumor clusters. Herein, we generated a transgenic murine model with red fluorescent lymphatics that, with implantation of tumor cells in a dorsal skin window chamber, simulate IBC characteristics. This model enables intravital imaging and quantitative analysis of collectively migrating tumor cells and vessel density in the local tumor microenvironment, an innovation that can be widely applied to various cancers exhibiting LVI.

**Abstract:**

Inflammatory breast cancer (IBC), an understudied and lethal breast cancer, is often misdiagnosed due to its unique presentation of diffuse tumor cell clusters in the skin and dermal lymphatics. Here, we describe a window chamber technique in combination with a novel transgenic mouse model that has red fluorescent lymphatics (ProxTom RFP Nu/Nu) to simulate IBC clinicopathological hallmarks. Various breast cancer cells stably transfected to express green or red fluorescent reporters were transplanted into mice bearing dorsal skinfold window chambers. Intravital fluorescence microscopy and the in vivo imaging system (IVIS) were used to serially quantify local tumor growth, motility, length density of lymph and blood vessels, and degree of tumor cell lymphatic invasion over 0–140 h. This short-term, longitudinal imaging time frame in studying transient or dynamic events of diffuse and collectively migrating tumor cells in the local environment and quantitative analysis of the tumor area, motility, and vessel characteristics can be expanded to investigate other cancer cell types exhibiting lymphovascular invasion, a key step in metastatic dissemination. It was found that these models were able to effectively track tumor cluster migration and dissemination, which is a hallmark of IBC clinically, and was recapitulated in these mouse models.

## 1. Introduction

Inflammatory breast cancer (IBC) is the most lethal variant of all clinically distinct types of breast cancer, accounting for 10% of breast cancer deaths despite its relatively rare occurrence [1]. By definition, all IBC tumors are at least stage III, with many patients exhibiting distant metastasis at diagnosis (30% compared to 5% for non-IBC patients) [2,3,4,5]. Even for IBC cases diagnosed without evidence of distant metastases, there is a higher rate of metastatic relapse [6,7]. Currently, there are no IBC-specific treatments; patients typically undergo an aggressive multidisciplinary treatment regimen, which includes surgery, endocrine therapy if hormone receptor-positive, radiation, chemotherapy, and targeted therapy, among other interventions [8,9,10,11]. Despite the recent advent of these multifaceted treatment approaches, 5-year survival for IBC patients is a dismal 40–50%, with a high incidence of tumor recurrence and metastatic progression [12]. Disproportionate rates of incidence and poor outcomes are also associated with self-identified African American women [13,14], recently linked to differential adaptive stress response gene expression [15]. Unlike other types of locally advanced breast cancers, the majority of IBC patients do not present with a solid tumor mass at diagnosis, nor is there any radiographic evidence of the presence of malignancy in the breast [16]. Instead, the clinicopathological hallmark of IBC is lymphovascular invasion (LVI) with the presence of tumor-cell clusters, termed tumor emboli, in the lymphovascular spaces [17,18,19,20]. These invasive tumor-cell clusters and lymphatic emboli are believed to underlie the inflamed appearance of the breast at clinical presentation, and their formation is postulated to be an efficient path to metastatic spread [5,17].

Studying IBC disease biology is profoundly impacted by the paucity of preclinical models that recapitulate these features of IBC progression. Traditional methods that measure tumor therapeutic response in vivo by monitoring tumor growth are biologically insufficient for investigating the diffuse spread of the IBC tumor cell clusters and lymphovascular emboli. In vitro, three-dimensional culture systems, such as tumor spheroids, mammospheres, and the IBC tumor emboli cultures that our previous studies have optimized for high content screening [20,21], provide a unique stage on which to study drug penetration and efficacy, local tumor invasion potential, and tumor-cell signaling. However, the in vitro culture systems have technical limitations due to variability in size, homogeneity of spheroids, and fragility of spheroid structure. Furthermore, there are imaging challenges with these preclinical models as they are unable to replicate the tumor microenvironment and vascular features of IBC [22]. Xenograft models using patient-derived IBC cell lines, such as SUM149, WIBC-9 and Mary-X (the latter both PDX-like, MDA-IBC3 models [21]) recapitulate lymphatic permeation and the LVI phenotype when tumor cells are co-implanted with mesenchymal stem cells to induce an invasive skin phenotype in the mouse [23]. However, these models provide limited quantitative information regarding the tumor microenvironment. There remains a critical need for models that allow for in vivo live monitoring and quantitative analysis of initial tumor growth and collective migration. In this present study, we have generated a transgenic murine model with red fluorescent lymphatic vasculature combined with the surgical implantation of tumor cells in the dorsal skin fold in a window chamber that simulates the IBC patient’s clinicopathological features of diffuse, collectively migrating tumor cells.

## 2. Materials and Methods

### 2.1. Cell Lines

Murine-derived, E0771 carcinoma cells (CH3 Biosystems, Amherst, NY, USA) were cultured in RPMI-1640 with 10% *v*/*v* fetal bovine serum. Murine-derived mammary-gland carcinoma cells, 4T1 with RFP stable expression previously prepared and described [24], were cultured in DMEM supplemented with 10% *v*/*v* of fetal bovine serum. The IBC, patient-derived SUM149 (cultured in Ham’s F-12, supplemented with FBS, insulin, hydrocortisone, HEPES, penicillin, and streptomycin, all purchased from Sigma Chemical Co., Burlington, MA, USA) and the SUM190 cells (cultured in serum-free medium with insulin and hydrocortisone) [25] used in this study have stable expression of the GFP reporter, as previously described [22,26]. The isotype-matched variant, SUM149shXIAP-GFP [26], was cultured in the same media as SUM149. The cell lines were authenticated at regular intervals by short tandem repeat polymorphism analysis at the Duke Sequencing Facility (Durham, NC, USA). All cell lines were cultured for less than two months before used in these studies at 37 °C, 5% CO_2_, and harvested by trypsinization at approximately 80% confluence in log phase growth before implantation.

### 2.2. Generation of Transgenic Mice with Red Fluorescent Lymphatic Vasculature

All animal studies were performed in accordance with protocols approved by the Duke University Institutional Animal Care and Use Committee (IACUC) and adhere to the NIH Guide for the Care and Use of Laboratory Animals. The C57/BL6 mice bearing the tdTomato (Tg, Prox1-tdTomato, 12Nrud) gene under control of a *Prox1* promoter (Jackson Laboratory, Bar Harbor, ME, USA) [27] were crossed with athymic nude mice at the Duke University Breeding Core Facility (Durham, NC, USA). *Prox1* encodes a transcription factor (prospero-related homeobox 1) that is necessary for the formation and maintenance of lymphatic vessels; as such, the transgenic mice (hereafter referred to as ProxTom RFP Nu/Nu mice) exhibit red fluorescent lymphatic vessels. The mice were housed in a germ-free environment at the Duke Laboratory Animal Resources (DLAR) and provided with food and water ad libitum.

### 2.3. Dorsal Window Chamber Surgery and Tumor Cell Transplantation

**Figure 1:**
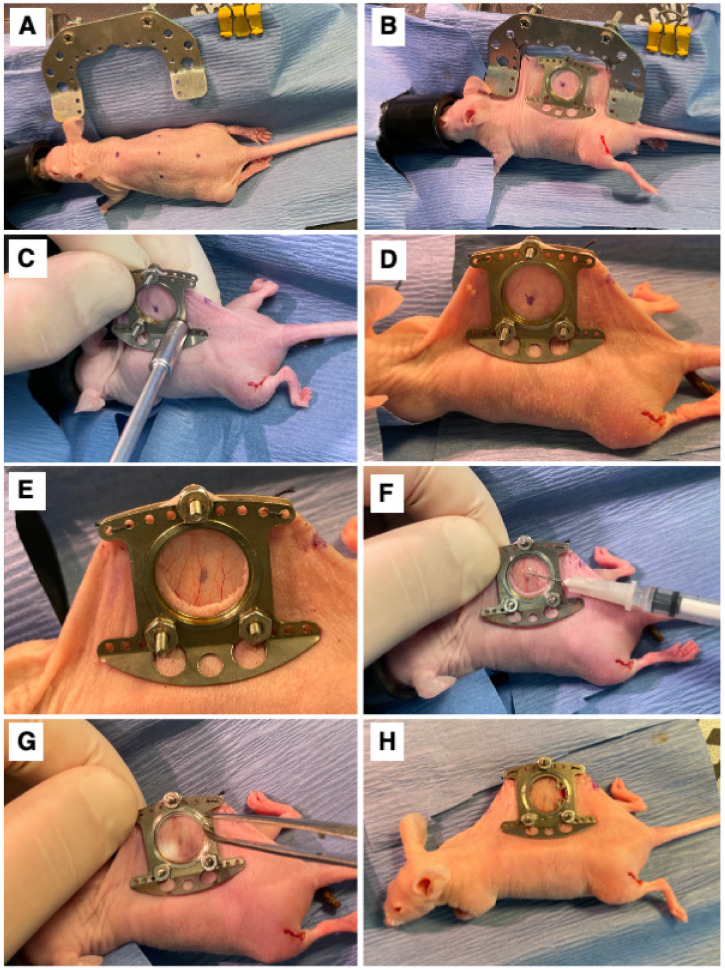
Surgical Procedure. (**A**) The marked center line and window view area on the mouse. (**B**) Surgical attachment of window chamber apparatus to dorsal skinfold of mouse. (**C**) Securing of window chamber to mouse. (**D**) Side view of window chamber attached to dorsal skinfold of mouse. (**E**) The exposed fascia after removal of skin inside the window view area. (**F**) Implantation of cells into exposed fascia of mouse. (**G**) Attachment of cover glass secured with a retainer ring on window chamber. (**H**) View of mouse after completed window chamber implantation surgery.

A dorsal skinfold window chamber was implanted in ProxTom RFP Nu/Nu mice [22,28]. Briefly, mice were anesthetized under 1–2% isoflurane (Patterson Veterinary Supply, Greeley, CO, USA) in 100% O_2_ carrier gas and placed on a heated pad to maintain an internal temperature of ~38 °C. Ophthalmic ointment was applied, and skin was prepped by wiping with 4% chlorhexidine and followed with 70% ethanol three times. The animals were wrapped in a sterile drape with the dorsal skin exposed. The mouse’s skin was tented and sutured to a c-frame to hold it in position (Figure 1A,B). Next, three, 1 mm diameter holes were cut through which the window frame could be secured (Figure 1C,D), and a 12 mm diameter skin punch was removed from the superior skin fold only (Figure 1E). The titanium window frame (Small Dorsal Kit, APJ Trading, Ventura, CA, USA) was sutured in place, and tumor cells, which included E0771 (1.0 × 10^5^), 4T1-GFP (7.5 × 10^4^), SUM149-GFP (1.0 × 10^5^), SUM190-GFP or dual GFP-Luc (1.0 × 10^5^), or MDA-IBC3-RFP (1.0 × 10^5^), suspended in 20µL of PBS, were injected under the fascia in the center of the window (Figure 1F). Sterile saline was used to fill the window space, over which a cover glass was placed and affixed with a retaining ring (Figure 1G). The animal was allowed to recover on a warming pad and was then returned to its original housing (Figure 1H). Buprenorphine SR (0.15 mg/kg) was administered subcutaneously for analgesia once a day for three days following surgery. Mice were monitored 3× weekly for weight loss. Mice were humanely euthanized using Euthasol at the end of the study (maximum 10 days post-surgery) or if the mouse experienced surgical complications. All animal experiments were approved and performed in accordance with guidelines from the Duke Institutional Animal Care and Use Committee (IACUC).

### 2.4. Intravital Imaging of Tumor Cell and Lymphatic Dynamics in the Mice Bearing Dorsal Window Chambers

**Figure 2:**
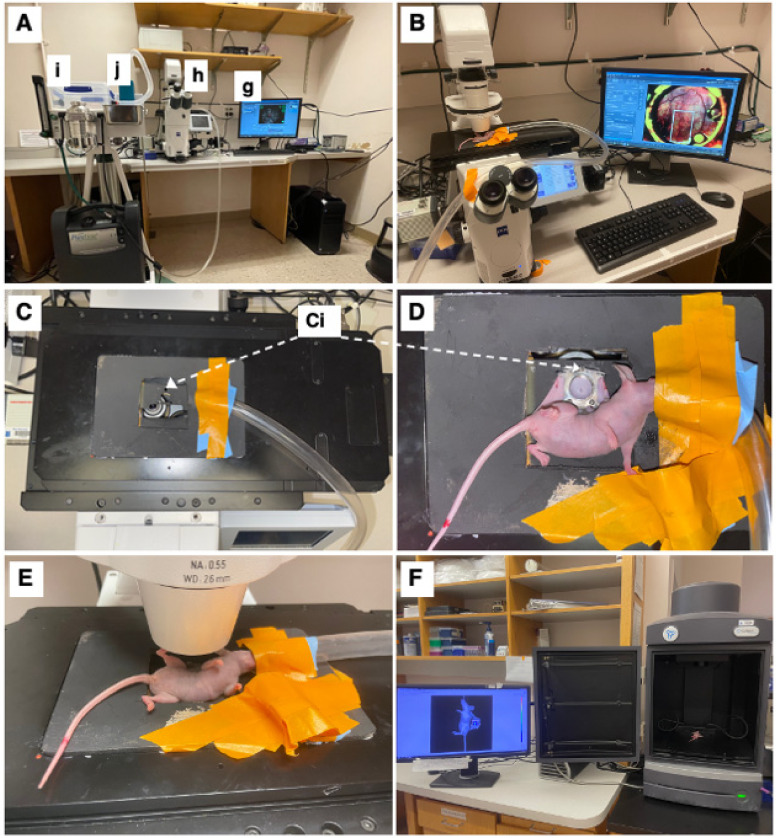
Intravital microscopy and imaging. (**A**) Equipment setup including a computer with appropriate imaging software (here ZEISS ZEN blue edition) (**g**), ZEISS Axio Observer inverted microscope (**h**), and an isoflurane station (**i**) with an isoflurane tube (**j**) connected to the microscope stage. (**B**) Top view of microscopy setup with imaging stage, with mouse fixated on imaging stage. (**C**) Top-view of imaging stage and chamber-to-platform ((**Ci**), Chamber-to-stage platform secured with metal nuts). (**D**) Top view of the mouse, fixated on imaging stage. (**E**) Side view of the mouse on the microscope stage. (**F**) IVIS imaging setup with window chamber implanted mouse (luciferase luminescence imaging shown).

The mice were imaged on an inverted microscope (Zeiss Axio Observer Z1, Carl Zeiss Microscopy, White Plains, NY, USA) at 5× magnification (Zeiss FLUAR 5×/0.25) using a 16-bit, air-cooled CCD array (Orca Flash 4.0, Hamamatsu, 2048 × 2048 pixels, Japan) (Figure 2B). As part of this microscope’s setup, a Quad-View image splitter (QV2, Photometrics, Tucson, AZ, USA) was in place between the camera and microscope but remained in bypass mode and was not used in this study. A mercury fluorescent light source (X-Cite 120Q, Excelitas Technology, Waltham, MA, USA) was used for reflectance measurements, and a halogen lamp was used for brightfield transmission images. For imaging of the GFP-labeled cells, a filter cube (Semrock, Rochester, NY, USA) with an excitation wavelength of 470/40 nm and an emission bandwidth of 525/50 nm was used. For imaging of the RFP-labeled lymphatics, a filter cube (Semrock, Rochester, NY, USA) with an excitation wavelength 562/40 nm and an emission bandwidth of 624/40 nm was used. Brightfield images were obtained under a polarized, epi-illumination filter (Chroma Technology Corp, Bellows Falls, VT, USA). Each mouse received a tiled image across its window chamber so that the entire skinfold was imaged. The exposure time was kept constant during these tiled images but varied across mice. Because the window was not always perfectly flat, a z-stack was used to obtain images across 500 mm that focused at 10 equally spaced planes. In the case of a notable area of interest, the apotome (Zeiss ApoTome.2, Carl Zeiss Microscopy) was used in small areas to obtain depth-resolved images. Zen Pro software (2012, Carl Zeiss Microscopy) was used to acquire all images.

The mice were anesthetized under 1–2% isoflurane (Patterson Veterinary Supply, Greeley, CO, USA) in a 100% O_2_ carrier gas for imaging (Figure 2A(i,j)). A heating pad set at 42 °C kept the mice warm (internal temperature around 38 °C) for the duration of each imaging session (~30 min/session). Ophthalmic ointment (LubriFresh P.M., Major Pharmaceuticals, Livonia, MI, USA) was placed on the eyes. A custom frame—the approximate size of a microscope slide—was bolted onto the extended bolts of the window chamber so that the window remained steady while imaging (Figure 2C,D). The mice were imaged at several timepoints (Figure 2E). On the day of surgery, the timepoints were somewhat inconsistent, ranging from 0 h to 6 h post-surgery. Mice were then imaged daily 5–10 days post-surgery when they were humanely sacrificed. The mice were also imaged using the in vivo imaging system (IVIS) (Perkin-Elmer, Waltham, MA, USA); this process was repeated every day starting with 6 h until day 6 (Figure 2F) to validate sustained tumor growth over the experimental time period.

### 2.5. Image Processing

Once the images were obtained, they were normalized to the exposure time using the Zen Pro software by dividing reference images into each tile. These newly normalized tiled images were exported into uncompressed .tiff. files and analyzed with FIJI [29] for the remainder of image processing and quantification. Once the 16-bit tiff. images were imported into FIJI, and they were converted to 8-bit images for a threshold analysis. The plugin “Stack Focuser” (open source, author Michael Umorin, https://imagej.nih.gov/ij/plugins/stack-focuser.html accessed 1 January 2020) was used to combine the z-stacked image into a single, in-focus image. The edges of the window were commonly at a different focus than the center, just as the deeper blood vessels are focused on a different plane than a bulging tumor. The stack focuser algorithm processes the multiple foci (or z-stack) such that the edges of the window and the center of the window are in focus in a single image. The kernel was set to 6. This was repeated for each channel: GFP, RFP, and brightfield.

### 2.6. Image Analysis

The tumor cell channel (GFP) was set at a threshold to include all pixels with greater than the mean ± 2 standard deviations of the background signal. This created a binary image where we quantified the positive fraction. Because some noise and artifacts were still present (for instance, a chip or scratch on the coverslip), a subjective number of erode and dilate functions were used until the noise was no longer contributing a signal, but the overall shape and signal area of the tumor clusters remained. This was repeated for each time point and each tumor cluster image. The total tumor cell area was quantified by summing the positive fraction of the binary image and then converting each pixel into millimeters. The “analyze particles” function in FIJI software was used to quantify (1) the number of tumor cell clusters and (2) the coordinates of each tumor cell cluster relative to the center of the mass. The function parameters were set to count any clusters greater than 50 pixels^2^ (~0.0013 mm^2^) and to avoid noise/artifacts from very small regions (<~0.001 mm^2^). Particles were not excluded based on circularity. Masks were created around each cluster, and these were used to quantify the number of clusters present as well as their linear distance to the center of the mass. The area of each mask was also computed, and the percent of total area was computed for each cluster. The area moment, which describes the basic directional growth pattern of the tumor cells, was quantified by multiplying a cluster’s distance from the center of the mass by its area. The diameter of each tumor cluster was approximated by assuming that each cluster was a circle and solving for the diameter using the area of the circular cluster. For each subject and each timepoint, the data was summarized by (1) the number of tumor clusters, (2) the total area, (3) the average area of each cluster (±SEM), (4) the average (±SEM) diameter of each cluster, and (5) the average (±SEM) area moment.

### 2.7. Quantifying Linear Lymph and Blood Vessel Density

To quantify the vascular density (vessels/mm) in FIJI, the GFP channel was used for visualizing blood vessels, and the RFP channel was used to visualize lymph vessels. A region of interest (ROI) was centered in the window, and its area was recorded. Within this ROI, the center line of easily visualized vessels was traced by hand, and their lengths were summed. The total length of vessels divided by the area of the ROI resulted in the linear vascular density of the vessels. This analysis was repeated for each timepoint per subject and for both blood and lymph vessels.

## 3. Results

### 3.1. Murine Models to Simulate IBC Clinical Presentation

**Figure 3:**
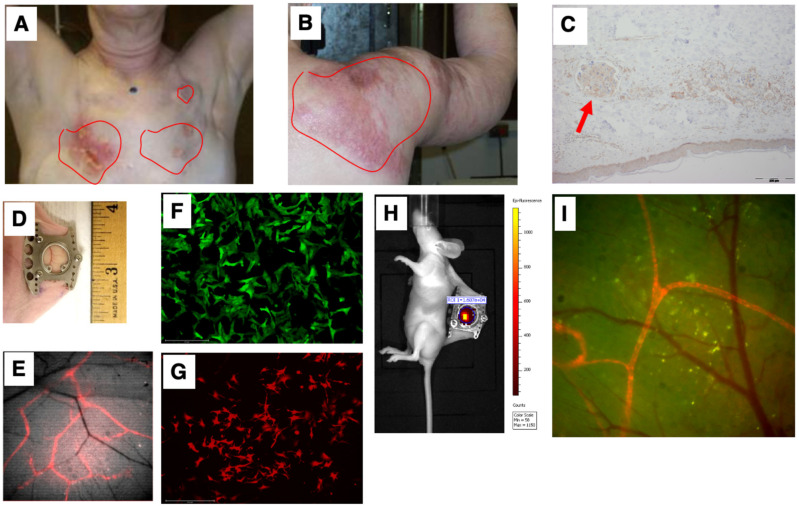
Simulation of the IBC clinicopathological characteristics. Representative images of deidentified IBC patients showing tumor cell clusters on (**A**) chest wall and (**B**) shoulder; (**C**) characteristic presence of tumor cell clusters (indicated by red arrow) in the lymphatic vessels (termed tumor emboli) of IBC; (**D**) representative image of dorsal skinfold window chamber; (**E**) red fluorescence in transgenic nude mice model (ProxTom RFP Nu/Nu mice); representative images (10×) of (**F**) SUM149-GFP IBC cells and (**G**) PDX derived MDA-IBC-3-RFP; (**H**) IVIS imaging of tumor cell fluorescence; and (**I**) visualization of tumor cell growth, motility, and/or vasculature by live fluorescent imaging of the window chambers.

Our first goal was to develop a preclinical model that simulated the unique IBC clinicopathological presentation of diffuse tumor cell clusters exhibiting dermal invasion (Figure 3A,B shows representative patient photomicrograph; Figure 3C shows the presence of tumor cell clusters termed tumor emboli in the lymphatic vessels). To this end, we employed the dorsal skinfold window chamber technique (Figure 3D) that allows for in vivo, microscopic examination of implanted tumor cells and the ability to track dynamic changes of the tumor in its local microenvironment from the time of implantation up to 2 weeks [28,30]. Furthermore, to specifically facilitate visualization of lymphatic and endothelial vessels along with tumor–vessel interactions, we generated a transgenic nude mice model (ProxTom RFP Nu/Nu mice; Figure 3E), wherein the mice exhibited red, fluorescent lymphatics (tdTomato fluorophore under control of a Prox1 promoter, which encodes a transcription factor, prospero-related homeobox 1) necessary for the formation and maintenance of lymphatic vessels. Next, we generated various GFP- or RFP-tagged breast cancer cells (representative images in Figure 3F,G) for subcutaneous implantation in the window chamber bearing athymic or ProxTom RFP Nu/Nu mice as needed and visualization of tumor cell growth, motility, and/or vasculature by live fluorescent imaging of the window chambers (Figure 3H,I).

### 3.2. Multichannel Imaging of IBC Tumor Cells and Lymphatics Demonstrates Co-Localization

**Figure 4:**
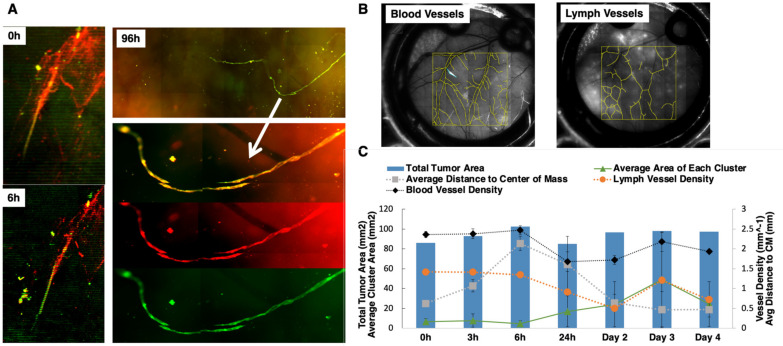
Characterization of tumor vessel interaction in the dorsal window chamber of Nu/nu ProxTom RFP transgenic mice. (**A**) Representative in vivo tumor vessel image confirming the presence of co-localized red and green fluorescence indicated by white arrow for lymphatic and tumor signal, the RFP channel monitors fluorescent lymphatics, and GFP channel images tumor cells. (**B**) Representative blood and lymph vessel density quantification. (**C**) Quantitation of tumor area, blood, and lymph density change over the course of 4 days. On the left axis, corresponding with the lines, the average area of each cluster (with standard deviation) shows an increasing trend. The average distance of each cluster of cells to the center of the mass (right axis) is decreasing over time. The blood vessel density and the lymph vessel density (right axis) show some decrease initially but no particular trend over time.

In order to track local tumor growth, tumor spread, and the lymph–tumor interactions of GFP-labeled SUM149 and SUM190 cells implanted in the ProxTom RFP Nu/Nu mice-bearing window chambers, we used multichannel optical microscopy over 4 days post-surgery. Representative, in vivo tumor vessel image (Figure 4A) confirms the presence of co-localized red and green fluorescence for lymphatic and tumor signal (channel overlap is mapped to yellow), respectively, and diffuse tumor cells along regions of lymphatic vessels both proximal and distal to the primary tumor site. Over a 96-h time course, we observed green tumor cells infiltrating the red lymphatic vessels. Next, we quantified how the blood and lymph vessel density changed over time by tracing the length of the vessels in a region of interest and then diving by region area to find density values (Figure 4B). The serially imaged window chambers with structural illumination were used to assess the total tumor signal without considering its spread (tumor area, right axis, Figure 4C). Graphical representation of the collected datasets show that the average tumor cluster area increased in size. Additionally, the distance of each disseminating tumor cell cluster from the center of the implanted mass within the window chamber decreased as the area moment (which describes the directional growth of the tumor) decreased (right axis). Finally, the vascular (blood and lymph) length and density showed some decrease over the course of 3 days but stabilized after day 4. Collectively, these quantitative parameters validated the use of this model to quantify local tumor growth, motility, and lymphatic invasion relevant to IBC phenotype.

### 3.3. Quantitative Analysis of In Vivo Motility of IBC Tumor Cell Clusters

**Figure 5:**
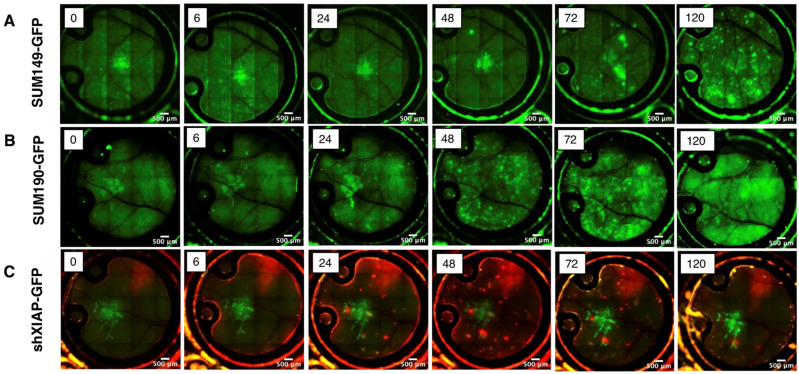
Time course of live imaging of the local tumor growth and migration in Nu/Nu or ProxTom mice of GFP-labeled (**A**) SUM149, (**B**) SUM190, and (**C**) shXIAPSUM149. Images at indicated time points; green fluorescence or red fluorescence; 5× magnification.

To further characterize IBC tumor cell growth and motility over time, in vivo imaging of the window chambers in mice implanted with cells were carried out over days 5–8 post-implantation. As shown in Figure 5, the basal-type, patient-derived SUM149 cell line (Figure 5A) appeared as a small mass in the center of the window chamber from the time of implantation (0 h through 48 h). However, by 72 h post-implantation, the tumor cells were observed to migrate away from the initial implantation site, becoming increasingly dispersed over time. The HER2 amplified, SUM190-GFP cell line exhibited similar chronological behavior, with tumor cell migration occurring as early as two days post-implantation and extending in some cases to the visible confines of the window chamber (Figure 5B). We used the shXIAP-GFP cell line, an isogenic derivative of SUM149 that has a stable knock-down of the anti-apoptotic protein, XIAP, as a control in this model. Similar to our previous report, wherein inhibition of XIAP expression inhibits IBC tumor cell motility [20], current data (Figure 5C) shows that the cluster of cells remain near the implantation site even after 5 days and do not spread like the parental SUM149 or SUM190 cells.

**Figure 6:**
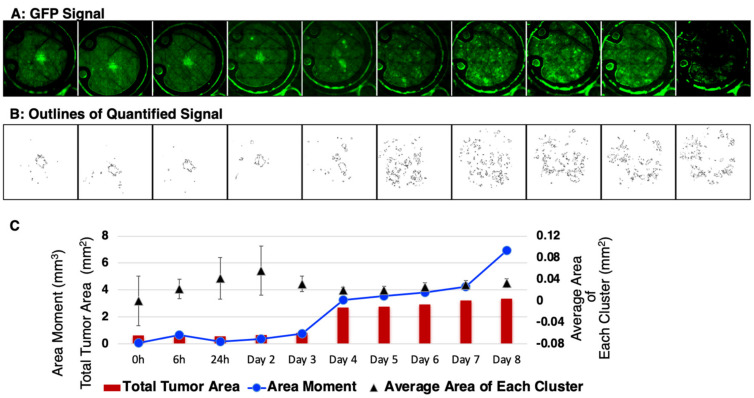
Quantitative analysis of the in vivo local tumor growth and migration. (**A**) Representative SUM149-GFP dorsal skinfold window chamber in a nude mouse shown (n = 5). (**B**) Masks created to show the threshold image of tumor signal corresponding to the tumor area in top panel. (**C**) Graphical representation of the calculated mean tumor area and the area moment describing the overall direction and movement of tumor-cell clusters.

Next, these datasets were subjected to quantitative analysis by setting the tumor cell channel (GFP) at a threshold to count any clusters greater than 50 pixels^2^ (~0.0013 mm^2^) and with greater than the mean ± 2 standard deviations of the background signal while avoiding noise/artifacts from very small regions (<~0.001 mm^2^) (Figure 6A). The representative data (Figure 6B) showed masks created around each tumor cell cluster. This allowed for the computation (Figure 6C) of the total tumor area including the average area of each cluster. In addition, the area moment, which describes the basic directional growth pattern of the tumor cells, was quantified by multiplying a cluster distance from the center of the mass by its area. Collectively, these results reveal the potential of this short-term, longitudinal imaging time frame in studying transient or dynamic events during tumor cell migration and reflect IBC patient characteristics.

## 4. Discussion

Understanding IBC tumor-cell progression presents unique challenges in terms of imaging and preclinical modeling as the tumor cells form clusters migrating collectively and diffusely through the skin and dermal lymphatics. In this current study, we generated a novel, transgenic mouse model exhibiting red, fluorescent lymphatics (ProxTom RFP nu/nu) combined with skin fold window chambers implanted with IBC tumor cells to assess local growth. This methodology utilized multichannel, in vivo imaging in the Nu/nu Proxtom RFP mice, which achieved spatial resolution and a field of view within the window chamber that was sufficient for quantitative analysis of cellular and vascular details in the tumor microenvironment and surrounding normal tissue. Notably, we quantified the distribution of tumor spread, which is unique to IBC. Other syngeneic or heterotopic models struggled to quantify the primary endpoint of tumor volume because of the particular method with which IBC migrates and grows. We also highlighted, through our structured-illumination imaging, that IBC cells potentially spread through lymphovascular invasion in in vivo models. This is a significant advancement in preclinical IBC modeling, as current in vitro studies use monolayer cells, mammospheres on Matrigel, 3D tumor spheroids, or tumor emboli cultures derived from IBC cell lines [20,31,32,33,34]. Three-dimensional cultures are limited due to spheroid heterogeneity and their inability to mimic the physical environment of the dermal lymphatic system. Furthermore, traditional fluorescent/bioluminescent subcutaneous or orthotopic mammary tumor implantation models to monitor tumor growth delay and metastatic progression [20,35,36,37,38] do not achieve sufficient resolution for measuring how tumor cells and tumor-cell clusters are interacting with the tumor microenvironment. Our data using breast cancer cell lines reveal that, similar to the hallmark clinicopathological presentation of tumor cell clusters in the dermal and lymphatic vessels in IBC patients, the IBC-patient, primary tumor-derived cell lines tested (SUM149 and SUM190) exhibited a diffuse and disseminating pattern within 3 days of implantation in the window chamber. Notably, this was unlike non-IBC cells that remained as a single tumor mass similar to previous reports [39,40] of non-IBC tumor growth in window chamber models (Supplementary Figure S1). Additionally, the structured-illumination imaging data demonstrates lymphovascular colocalization of the tumor cells with the lymphatics. This is a key, novel finding of this study and indicates that lymphovascular invasion may occur very early on in these models. This also suggests that this model may be useful in understanding the interplay between IBC tumor cells and the microenvironment and evaluating treatments designed to target the microenvironment or LVI characteristic of IBC tumors in patients.

One of the strengths of the dorsal window chamber model is that it is easily accessible for live imaging of the mouse over different time points. This model can be adapted to surgically implant a window in the mammary fat pad [39] of the Nu/nu ProxTom RFP mice for orthotopic tumor cell growth studies. The generation of the transgenic Nu/nu ProxTom RFP mice model has significant potential to supplement and improve current in vivo models of IBC growth and metastasis. For example, MARY-X, a transplantable, triple-negative IBC xenograft that exhibits lymphovascular invasion and formation of tumor emboli in vivo, has been used to probe the molecular and cellular mechanisms underlying the aggressive IBC phenotype for 20 years [21]. Nu/nu ProxTom RFP mice can be used for implantation of MARY-X in the ventrolateral flank or in the mammary fat pad [41] for quantitative optical imaging, as described in the current studies. This model could confirm the extent of lymphovascular invasion and has the potential to demonstrate how early interventions could limit metastases.

A major challenge with dorsal window chambers is rapid degradation over time. Windows do not last more than 2 weeks before the image quality degrades, obscuring microscopic details through optical scattering and attenuation. We have been challenged in accurately quantifying microscopic changes such as vascular density after postoperative day four. The tumor emboli, which are bright and large on imaging, can easily be quantified over many days (we quantified through day 8). We hope for future studies to include ex vivo histopathological analysis to further support our early tumor microenvironment changes.

Recent work suggests that mesenchymal stem cells (MSCs) and macrophages play an important role in recreating the IBC phenotype observed in patients [23,35,42]. Specifically, the co-injection of SUM149 mammospheres with human MSCs suppresses initial (day 0–6) tumor formation but promotes a pro-invasive mesenchymal phenotype, spontaneous metastasis, and dermal lymphovascular invasion in preclinical models. However, this model remains limited, as dermal invasion of the injected mammospheres cannot be visualized until after the study endpoint and tumor resection processes occur [35]. This limitation may be overcome by using the dorsal chamber window model in conjunction with Nu/nu ProxTom RFP mouse to evaluate in vivo the interaction of the tumor cells with emboli and MSC co-injection on IBC lymphovascular invasion and metastasis. Alongside visually displaying tumor–lymphatic interactions, skin and tumor samples from the window chamber may be used in immunohistochemistry and other immune-profiling techniques. In addition, our data (shXIAP-GFP; Figure 5C) show that the knock down of XIAP (inhibitor of programmed cell death), implicated in aggressive IBC progression [22,43,44], suppressed migration in the window chamber model compared to an isogenic-matched parental IBC cell line. This shows the strength of this in vivo tumor model in drug discovery. We envision treating tumor cells with agents either pre-implantation or by local delivery near the tumor-implanted site to evaluate the effect on tumor cluster formation and collective migration/invasion patterns. This short-term model could allow for low-cost, rapid screening of agents, allowing for top candidates to be selected for studies using traditional, long-term tumor growth delay and metastatic models. We have demonstrated that multichannel imaging is a viable imaging paradigm for two fluorescent sources; additional fluorescent probes would provide valuable insight into the complex interactions between IBC, the lymphovascular system, MSCs, or other aspects of the microenvironment.

## 5. Conclusions

In summary, the imaging methodologies developed, along with the generation of the Nu/nu ProxTom RFP mice, provides a platform for quantitative measurements of locoregional invasion, tumor cell-lymphatic, or endothelial vessel interactions of implanted tumor cells or tumor emboli. The transgenic lymphatic mice model and the in vivo imaging algorithms developed have the potential to be widely applicable to study tumor–vessel interactions and invasion in other cancers and for testing drugs targeting LVI.

## Data Availability

The data presented in this study are available in the article and supplementary material.

## Supplementary Materials

The following supporting information can be downloaded at: https://www.mdpi.com/article/10.3390/cancers15082261/s1, Figure S1: Representative green or red fluorescence images (5×) at day 5 of murine non-IBC breast cancer cell lines used as controls (A) 4T1-RFP; (B) E0771 (dark mass depicted within green circle, under bright field); (C) representative image showing position of window chamber without tumor cells.
