# Supplementary material for: A Novel Preclinical Murine Model to Monitor Inflammatory Breast Cancer Tumor Growth and Lymphovascular Invasion
Source: Cancers (Basel). 2023 Apr 12;15(8):2261. doi: 10.3390/cancers15082261 (PMC10137020; doi:10.3390/cancers15082261)
Supplement: Supplementary file 1 [file cancers-15-02261-s001.zip › cancers-2234965-supplementary.pdf]

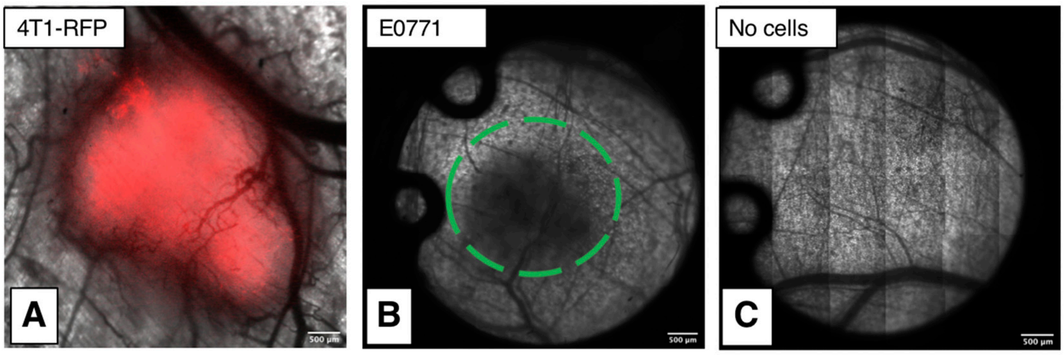

Supplemental Figure S1. Representative green or red fluorescence images (5X) at day 5 of murine non-IBC breast cancer cell lines used as controls (A) 4T1-RFP, (B) E0771 (dark mass depicted within green circle, under bright field), (C) representative image showing position of window chamber without tumor cells.
